# BAP1 promotes viability and migration of ECA109 cells through KLF5/CyclinD1/FGF‐BP1

**DOI:** 10.1002/2211-5463.13105

**Published:** 2021-03-28

**Authors:** Fengyun Wang, Ming Luo, Honglan Qu, Yufeng Cheng

**Affiliations:** ^1^ Cheeloo College of Medicine Qilu Hospital ShanDong University China; ^2^ The Third Affiliated Hospital of BaoTou Medical College Mongolia China; ^3^ Inner Mongolia Agricultural Hospital YaKeShi China

**Keywords:** BAP1, CyclinD1, esophageal carcinoma, FGF‐BP, KLF5

## Abstract

More than 40 000 patients worldwide die from esophageal cancer annually. The 5‐year survival rate of patients is only ~ 15–20%, and thus, there is an ongoing need to improve diagnosis and treatment of esophageal cancer. Breast cancer type 1 susceptibility protein (BRCA1)‐associated protein (BAP1) is a marker of poor prognosis in several cancers, including uveal melanoma, renal cell carcinoma, cholangiocarcinoma, non‐small cell lung cancer, and colorectal cancer. BAP1 mutations are early and rare events in esophageal carcinoma, but the involvement of BAP1 in progression of esophageal carcinoma is unclear. Here, we report that cell proliferation and migration were significantly enhanced in esophageal carcinoma ECA109 cells overexpressing BAP1, while they were diminished upon BAP1 knockdown. In addition, the expression of Krüppel‐like factor 5 (KLF5), CyclinD1, and FGF‐BP1 was increased by BAP1 overexpression and decreased by BAP1 knockdown. Our data suggest that BAP1 promotes cell proliferation and migration, and enhances the expression of KLF5 and its downstream genes, including CyclinD1 and FGF‐BP1, in the esophageal carcinoma cell line ECA109.

## Graphical abstract

Overexpression of BAP1 in ECA109 results in enhanced cell proliferation and migration and increased expression of Krüppel‐like factor 5 (KLF5) and its downstream genes. Knockdown of BAP1 in ECA109 results in a decrease in cell proliferation and migration, as well as a reduction in the expression of KLF5 and its downstream genes.

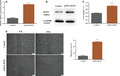

**Abbreviations**
BAP1: breast cancer type 1 susceptibility protein‐associated protein
BRCA1: breast cancer type 1 susceptibility protein
FGF‐BP1: fibroblast growth factor‐binding protein 1
KLF5: Krüppel‐like factor 5

Esophageal carcinoma is one of the most serious malignant tumors worldwide. According to statistics, more than 40 000 patients worldwide die from esophageal cancer annually. Esophageal cancer is the eighth most common tumor type and the sixth most common cause of tumor‐related deaths worldwide. Based on the 2015 cancer incidence statistics in China, 477 900 new patients were diagnosed with esophageal cancer and 375 000 patients died from esophageal carcinoma [1]. Despite the rapid development of medical technology in the past 10 years, and the significant progress in the diagnosis and treatment of esophageal cancer, the 5‐year survival rate of patients is only ~ 15–20% [2]. Atypical symptoms in the early stages of esophageal cancer and the lack of early warning signs indicate that patients are typically diagnosed with esophageal cancer in the advanced stages. Therefore, studying the pathogenesis of esophageal cancer is important for the early diagnosis and treatment of esophageal cancer.

Breast cancer type 1 susceptibility protein (BRCA1)‐associated protein (BAP1) is encoded by the corresponding gene located on chromosome 3p21 and is the only member of the ubiquitin carboxyl‐terminal hydrolase family that is localized to the nucleus and has low enzymatic activity [3, 4]. BAP1 is closely related to the development of many tumors and mainly acts as a tumor suppressor. The overexpression of BAP1 in the breast cancer MCF‐7 cell line inhibits cell colony formation [5]. The overexpression of wild‐type BAP1 in lung cancer NCI‐H226 cells can significantly inhibit the tumorigenic ability of cells in nude mice [6]. Deletions, loss of heterozygosity, missense mutations, and large rearrangements in the BAP1 gene locus have been found in lung and sporadic breast tumors and lung cancer cell lines [7]. In addition, BAP1 can remove the ubiquitination modification of the substrate protein, allowing the substrate to escape the ubiquitin–proteasome degradation pathway and enhance its stability, or affect the functional activity of the substrate, thereby regulating the related signaling [8]. It has been shown that BAP1 promotes the development of breast cancer by releasing the ubiquitin modification of the transcription factor human Krüppel‐like factor 5 (KLF5) [9]. However, whether BAP1 regulates KLF5 expression in esophageal cancer is unclear.

We measured the cell proliferation and migration potential of BAP1 overexpression and knockdown systems. We also examined the expression of KLF5 and its downstream target genes CyclinD1 and FGF‐BP1 at the mRNA and protein levels.

## Materials and methods

### Cell line and culture

Cells from ECA109, an esophageal carcinoma cell line, were provided by the Chinese Academy of Sciences (Shanghai, China). ECA109 cells were cultured in RPMI1640 (Thermo Fisher Scientific, Waltham, MA, USA) supplemented with 10% FBS at a constant temperature of 37 °C in a humidity chamber with 5% CO_2_.

### Plasmids, siRNA, and cell transfection

The PcDNA‐BAP1 plasmid and empty vector were purchased from OBIO Technology (Shanghai, China), and siRNA‐BAP1 or NC was provided by Genepharm (Shanghai, China). The cell culture medium was replaced with serum‐free medium 24 h before transfection. Plasmid or siRNA was mixed with serum‐free culture medium and maintained for 5 min (mixture A). Lipofectamine 2000 was diluted with serum‐free culture medium (mixture B). Mixture B was then added to mixture A and maintained for 10 min (mixture A + B). When the cells reached 60% confluence, mixtures A and B were gently added to the cells and the cells were cultured for 24 h. Cells were harvested at 24 h post‐transfection and used for further studies. For the BAP1 overexpression system, the empty vector was used as a control. For the BAP1 knockdown system, si‐NC was used as a control.

### MTT assay

After cell counting, the cell density was adjusted to 1 × 10^5^ cells/mL by dilution with serum‐free medium. One hundred microliters of cells per well was added to a 96‐well plate and cultured for 48 h. For the MTT assay, MTT was dissolved in PBS to a final concentration of 5 mg·mL^−1^; 10 μL was added to the cells and incubated further for 4 h. The OD value was recorded at 490 nm using a microplate reader (BMG CLARIOstar). The value of the control group was normalized to the average value, and the percentage of survival in other groups was calculated by dividing the ratio by the control average.

### Wound‐healing assay

A ruler, marker pen, and 6‐well plates were prepared and sterilized under ultraviolet light. Horizontal lines were drawn across the wells using a marker pen and ruler on the back of the 6‐well plate, and every well was crossed at least five times. Cell suspensions at a concentration of 1 × 10^6^ cells were prepared, and 1 × 10^5^ cells were added to each well. Vertical lines were drawn using a small pipette and a ruler. Cells were then washed three times with PBS. Floating cells were removed, and serum‐free medium was added. The cells were cultured in an incubator at 37 °C with 5% CO_2_ for 24 h and then photographed.

### Real‐time quantitative polymerase chain reaction (qRT–PCR)

RNA was extracted from the cells using TRIzol Reagent (Invitrogen, Carlsbad, CA, USA) according to the manufacturer's instructions, and RNA concentration was measured using a NanoDrop spectrophotometer. Genomic DNA was removed, and reverse transcription of 1 μg RNA was performed using the Transcriptor First‐Strand cDNA Synthesis Kit (Roche Applied Science, Basel, Switzerland). The expression levels of all genes, including BAP1, KLF5, CyclinD1, and FGF‐BP1, were measured at the mRNA level using the ABI 7500 Fast Real‐Time PCR System (Invitrogen). The $2^{‐\Delta \Delta C_{t}}$ method was used to calculate relative levels.

### Western blotting

After the cells were lysed, total protein was extracted. Protein quantification was performed using a BCA Kit, and SDS/PAGE was conducted. The protein from the SDS/PAGE gel was transferred onto a polyvinylidene difluoride membrane. The membrane was blocked using 5% nonfat dried milk for 2 h at room temperature. Primary antibodies were used for treatment at 4 °C overnight, and the membranes were then rinsed three times using PBST. Antibodies against BAP1 (ab245391, 1 : 2000), KLF5 (ab137676, 1 : 1000), CyclinD1 (ab134175, 1 : 10 000), and FGF‐BP1 (ab215353, 1 : 500) were provided by Abcam (Cambridge, UK). After washing again, the membranes were treated with the corresponding secondary antibodies and then washed three times using PBST. The protein bands were visualized using an ECL Kit and photographed.

### Statistical analysis

Data are presented as average ± SD values. The *t*‐test was applied using spss version 20.0 (SPSS Inc., IBM, Chicago, IL, USA). A *P*‐value < 0.05 (two‐sided) suggested a significant difference. Relative expression levels were plotted using graphpad prism 5 software (GraphPad Software; San Diego, CA, USA).

## Results

### Overexpression of BAP1 enhanced cell proliferation and migration in ECA109 cells

**Fig. 1:**
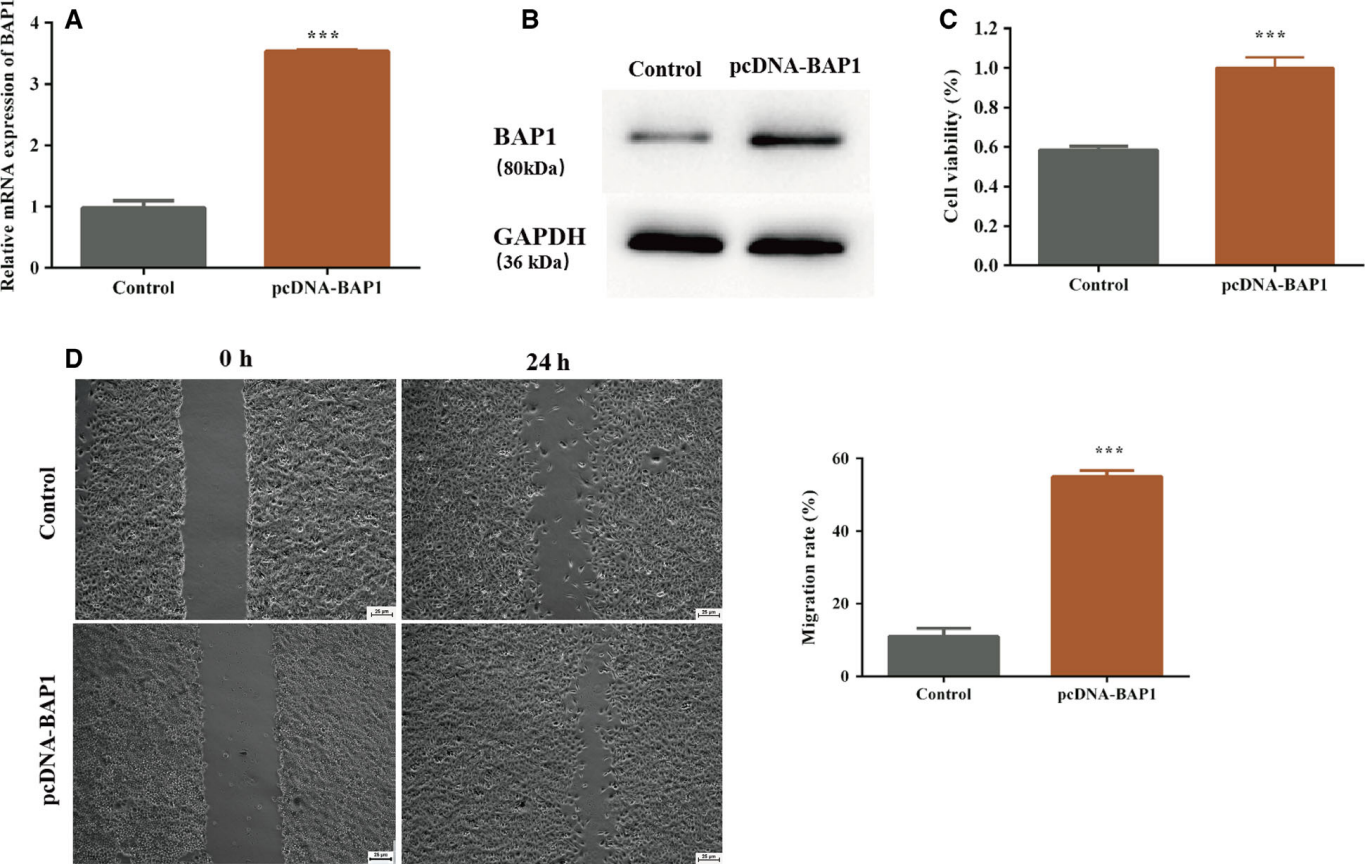
Overexpression of BAP1 enhances cell proliferation and migration in ECA109 cells. The control group included ECA109 cells transfected with the empty vector. (A) BAP1 mRNA expression in the pcDNA‐BAP1 and control groups; (B) BAP1 protein level in the pcDNA‐BAP1 and control group analyzed by western blotting; (C) cell viability of ECA109 cells in the pcDNA‐BAP1 and control groups; (D) cell migration of ECA109 cells in the pcDNA‐BAP1 and control groups (***P < 0.001 vs. control, Student's *t*‐test). The error bars indicate SEM. *N* = 3. Scale bar = 25 µm.

ECA109 cells were transfected with pcDNA‐BAP1 or an empty vector to induce the overexpression of BAP1. The efficiency of transfection results for pcDNA‐BAP1 showed that the expression of BAP1 was increased compared with that of the control (Fig. 1A). Western blot analysis of the protein expression level of BAP1 confirmed the overexpression of BAP1 in the pcDNA‐BAP1 group (Fig. 1B). The MTT assay was used to evaluate cell proliferation, and the results revealed that cell proliferation was notably enhanced in ECA109 cells transfected with pcDNA‐BAP1 compared to those transfected with the empty vector (Fig. 1C). Cell migration was then evaluated by the wound‐healing assay, and the results suggested that the migration of ECA109 cells transfected with pcDNA‐BAP1 increased notably compared with that in the control (Fig. 1D).

### Knockdown of BAP1 impeded cell proliferation and migration in ECA109 cells

**Fig. 2:**
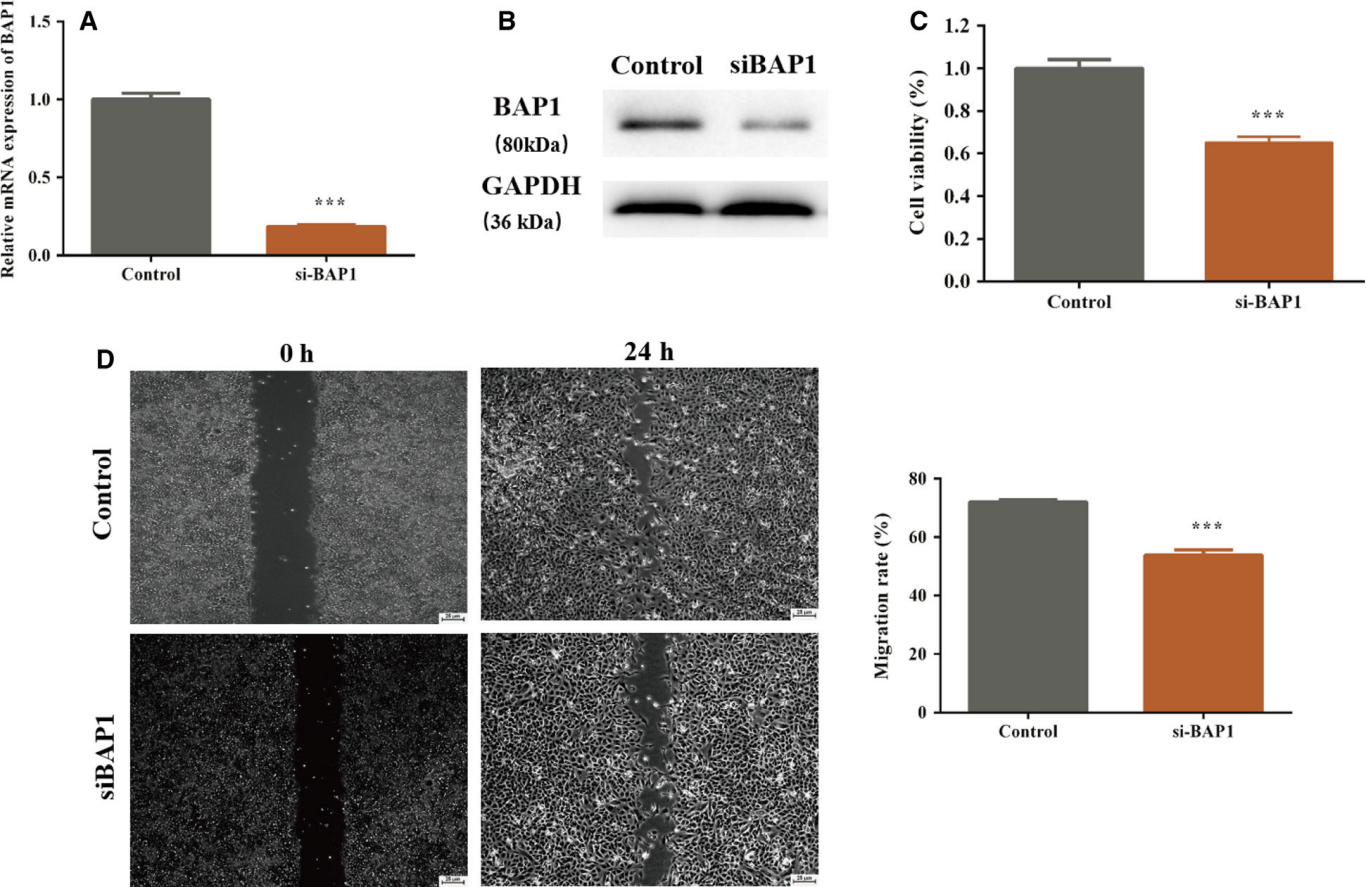
Knockdown of BAP1 impedes cell proliferation and migration in ECA109 cells. The control group included ECA109 cells transfected with si‐NC. (A) BAP1 mRNA expression in the si‐BAP1 and control groups; (B) BAP1 protein level in the pcDNA‐BAP1 and control groups analyzed by western blotting; (C) cell viability of ECA109 cells in the si‐BAP1 and control groups; (D) cell migration of ECA109 cells in the si‐BAP1 and control groups (***P < 0.001 vs. control, Student's *t*‐test). The error bars indicate SEM. *N* = 3. Scale bar = 25 µm.

To further examine the effect of BAP1 on cell proliferation and migration, knockdown of BAP1 was achieved using siRNA‐BAP1. The efficiency of transfection results for siRNA‐BAP1 showed that the expression of BAP1 was reduced in the siRNA‐BAP1 group compared with the NC group (Fig. 2A,B). Similar to the overexpression of BAP1, cell proliferation and migration were measured by MTT and wound‐healing assays in the BAP1 knockdown system. The results showed that cell proliferation and migration in ECA109 cells transfected with siRNA‐BAP1 were notably inhibited compared with that in the NC group (Fig. 2C,D).

### BAP1 expression affected KLF5 and its downstream genes, including CyclinD1 and FGF‐BP1

**Fig. 3:**
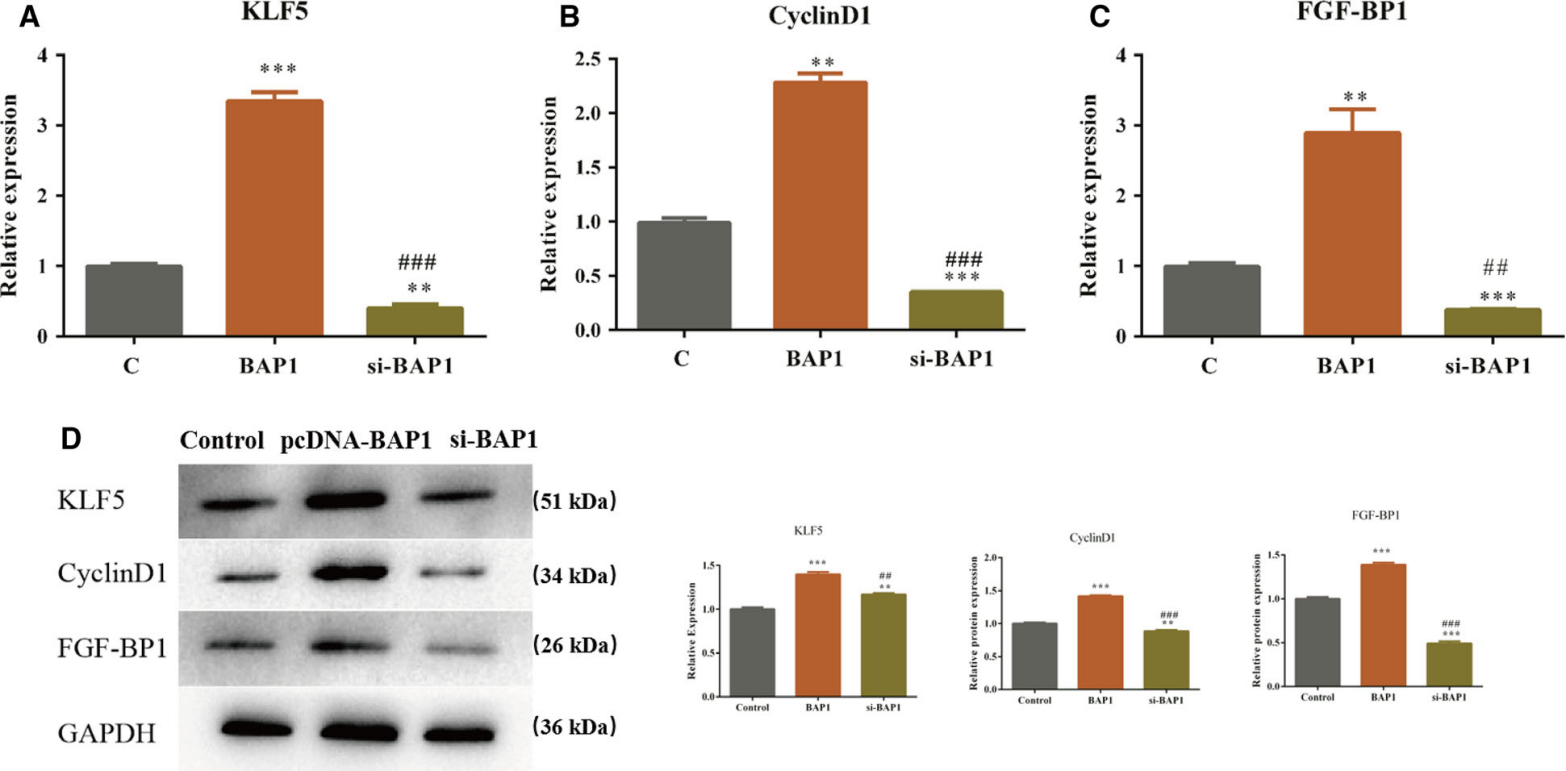
The expression of BAP1 affects KLF5 and its downstream genes including CyclinD1 and FGF‐BP1. (A) mRNA expression of KLF5, and its downstream genes CyclinD1 and FGF‐BP1, in the BAP1 overexpression and knockdown systems; (B) protein levels of KLF5, and its downstream genes CyclinD1 and FGF‐BP1, in the BAP1 overexpression and knockdown systems (**P < 0.01, ***P < 0.001 vs. control; ^##^ *P* < 0.01, ^###^ *P* < 0.001 vs. pcDNA‐BAP1, Student's *t*‐test). The error bars indicate SEM. *N* = 3.

Qin *et al*. [9] reported that BAP1 interacts directly with KLF5 in breast cancer and that KLF5 is stabilized by deubiquitinase BAP1. In our study, we investigated whether KLF5 expression would change when the expression of BAP1 increased or reduced. As expected, the expression of KLF5 was enhanced in the BAP1 overexpression system compared with that in the control (Fig. 3A). KLF5 mRNA levels were also significantly reduced in ECA109 cells transfected with siRNA‐BAP1 compared with those in the NC group (Fig. 3A). Interestingly, we found that the expression of downstream genes, KLF5, CyclinD1, and FGF‐BP1, was positively regulated by the expression of KLF5. As shown in Fig. 3A, the expression of CyclinD1 and FGF‐BP1 reduced or increased significantly in the BAP1 knockdown or overexpression systems, respectively. In addition, the expression of KLF5, CyclinD1, and FGF‐BP1 was further verified at the protein level and showed similar tendencies to the mRNA expression levels (Fig. 3B).

## Discussion

The incidence of esophageal cancer in China is considerably high, second only to that of gastric cancer [10]. Particularly in rural areas of China, esophageal carcinoma has one of the highest incidences among all tumor types [11]. The early surgical resection rate of esophageal cancer is 100%, and the 5‐year survival rate is more than 90%. However, most patients are diagnosed with esophageal cancer in the middle and advanced stages, and the 5‐year survival rate after surgical resection is still relatively low. Therefore, the identification of markers for the early diagnosis and prognosis of esophageal carcinoma is of great significance in clinical practice.

In recent years, BAP1 has been studied extensively in various cancers. Luchini *et al*. first reported a meta‐relationship between BAP1 deletion and the diagnoses and prognoses of various cancer types, and confirmed that BAP1 is a marker of poor prognosis in diverse cancer types, including uveal melanoma, renal cell carcinoma, cholangiocarcinoma, non‐small cell lung cancer, and colorectal cancer [12]. Inactivating BAP1 mutants were found in two patients with uveal melanoma by exon capture and large‐scale sequencing, and BAP1 mutants were also found in 25 (45%) of 55 additional cases of uveal melanoma [13]. Andrici *et al*. suggested that the deletion of BAP1 is a diagnostic marker of mesothelioma in effusion cytology [14]. However, BAP1 has rarely been studied in esophageal cancer. BAP1 mutations have been reported as early and rare events in esophageal cancer [15].

In this study, we aimed to explore the function of BAP1 in the pathogenesis of esophageal squamous cell carcinoma. Qin *et al*. [9] found that BAP1 promotes proliferation and metastasis in the breast cancer cell line MDA‐MB‐468. In our study, BAP1 was overexpressed and knocked down *in vitro*, and the proliferation and migration abilities of the cells were examined. MTT and wound‐healing assay results showed that the overexpression of BAP1 significantly enhanced cell proliferation and migration of ECA109 cells. Proliferation and migration of ECA109 cells were significantly inhibited by BAP1 knockdown, which is consistent with the results reported by Qin *et al*. [9].

In our study for determining the mechanism underlying BAP1 function, we occasionally observed that KLF5 expression correlated positively with BAP1 expression. Both mRNA expression and protein expression of KLF5 increased significantly in the BAP1 overexpression system, whereas the expression was impaired in the BAP1 knockdown system. KLF5 belongs to the SP/KLF transcription factor family and has three common C2H2‐type zinc finger structures at the carboxy terminus. Studies have shown that KLF5 can regulate the expression of a variety of genes, including CyclinD1, inducible nitric oxide synthase, plasminogen activator inhibitor 1, TGF‐β, and VEGF receptor genes [16]. In addition, Zheng *et al*. [17] have reported that KLF5 promotes breast cell proliferation by upregulating the transcription of fibroblast growth factor‐binding protein 1 (FGF‐BP1). We hypothesized that BAP1 expression may also affect target genes downstream of KLF5, such as CyclinD1 and FGF‐BP1. Next, we examined the mRNA and protein expression levels of CyclinD1 and FGF‐BP1 genes in the BAP1 overexpression and knockdown systems. We found that CyclinD1 and FGF‐BP1 were upregulated when BAP1 was overexpressed, while the expression levels of CyclinD1 and FGF‐BP1 were significantly reduced in the BAP1 knockdown system, which is consistent with the results reported by Zheng *et al*. [17].

## Conclusion

The overexpression or knockdown of BAP1 enhanced or impaired cell proliferation and migration, respectively, in ECA109 cells. Additionally, the expression of KLF5, CyclinD1, and FGF‐BP1 altered correspondingly. We concluded that BAP1 might promote cell proliferation and migration via KLF5/CyclinD1/FGF‐BP1 in ECA109 cells.

## Conflict of interest

The authors declare no conflict of interest.

## Author contributions

FW and YC conceived and designed the project. ML conducted the experiments. HQ analyzed and interpreted the data. FW wrote the manuscript.

## Data Availability

The research data are available from the corresponding author upon reasonable request.
