# Erratum to: BAP1 promotes viability and migration of ECA109 cells through KLF5/CyclinD1/FGF‐BP1

**DOI:** 10.1002/2211-5463.13313

**Published:** 2021-10-25

In the paper by Wang et al. [1], the affiliation for Fengyun Wang was incorrect. The correct affiliation is given below:

Qilu Hospital, Cheeloo College of Medicine, Shangdong University, China.
